# In Situ Real-Time Tracing of Organophosphorus Pesticides in Apples by Solid-Phase Microextraction with Developed Sampling-Rate Calibration

**DOI:** 10.3390/molecules24244444

**Published:** 2019-12-04

**Authors:** Xiao-Fan Zhang, Li-Li Zhao, Ming-Quan Huang, Xiu-Juan Li, Si-Yi Pan

**Affiliations:** 1 Key Laboratory of Environment Correlative Dietology (Ministry of Education), College of Food Science & Technology, Huazhong Agricultural University, Wuhan 430070, China; xiaofanzhang2010@163.com (X.-F.Z.); hzauzjy@163.com (L.-L.Z.); pansiyi@mail.hzau.edu.cn (S.-Y.P.); 2 Institute of Agricultural Products Processing, Henan Academy of Agricultural Sciences, Zhengzhou 450002, China; 3 China Light Industry Key Laboratory of Liquor Quality and Safety, Beijing Technology and Business University, Beijing 100048, China; huangmq@th.btbu.edu.cn

**Keywords:** in situ tracing, solid-phase microextraction, matrix compatibility, sampling-rate calibration, organophosphorus pesticides, apple

## Abstract

An in situ tracing study based on solid-phase microextraction (SPME) was conducted to investigate the uptake and elimination of organophosphorus pesticides in apples. A matrix-compatible polydimethylsiloxane/poly(styrene-co-divinylbenzene)/polydimethylsiloxane fiber was produced to meet the needs of in situ sampling. The fiber had high extraction ability, good sensitivity and accuracy with respect to the analytes in apple pulp, and could be used 85 times. Although the sampling rate was changing over time, quantification was still achieved by the sampling rate calibration method. Some factors that affect its applicability were studied. The limits of detection were 0.18 ng/g for diazinon and 0.20 ng/g for chlorpyrifos, rather lower than the maximum residue limits of the National Food Safety Standard of China (GB 2763-2016) and the European Commission (Reg.(EU) No 834/2013, 2018/686). The accuracy of in situ SPME quantification was verified by comparing with the results obtained by the traditional liquid–liquid extraction method. In this work, the in situ sampling method is developed using apples, diazinon, and chlorpyrifos as a model system; however, this method can be used for in vivo analysis of fruits and vegetables for nutrition and safety monitoring.

## 1. Introduction

The development of microsampling tools that allow for real-time monitoring of samples including living systems in natural environments is currently an important research topic in analytical chemistry. It is especially necessary for living subjects and unstable compounds because traditional analytical methods damage the samples and involve multifarious pre-treatment steps, which could affect the number and concentration of targeted compounds due to metabolism, stress response, biochemical reactions, and sample loss. Solid-phase microextraction (SPME) is one of the techniques applicable for in vivo/in situ analysis by integration of sampling, sample preparation, and sample introduction into one step using a syringe-like device [1,2]. It has been widely accepted as a convenient and sensitive sample preparation/enrichment technique for in vitro analysis of analytes from a broad range of samples that encompassing food, environmental, and biological matrices [3,4,5]. However it has been in recent years that in vivo SPME sampling has been developed and applied. Up to now, in vivo SPME sampling has been used for tracing pollutants [6,7,8], drugs [9], flavonoids [10], neurotransmitters [11], lipidomics [12], and so on in living systems and biological organisms. The major advantages of in vivo/in situ SPME are that it involves: (1) fast, simple, and green sample pre-treatment using a small portable fiber in a few minutes or for no more than 30 min, with the fiber able to be custom-tailored to meet specific requirements; (2) minimal perturbations to the system under study, able to accurately indicate what is happening and what will happen in the real world; and (3) repeated temporal and longitudinal studies using one experimental sample, which can reveal exposure history, uptake, and elimination of targets. Although remarkable progress has been made, the application of in vivo/in situ SPME is definitely not easy. Challenges include the development of matrix compatible coatings and establishment of quantitative calibration methods.

During the in vivo/in situ analysis, no sample pretreatment can be done and the SPME fiber is directly inserted in the sample. Owing to the complex matrix effect, the coating is easily polluted. To address this issue, a matrix-compatible coating was proposed by Souza Silva and co-workers [13]. It was accomplished by application of an external layer of polydimethylsiloxane (PDMS) over a commercial PDMS/divinybenzene (PDMS/DVB) fiber. PDMS is selected as a nonporous liquid coating, suffering less from the irreversible fouling effect caused by the complex matrix components when compared to solid coatings. The developed PDMS/DVB/PDMS fiber combines the benefits of the high sensitivity exhibited by PDMS/DVB and the robustness of PDMS. It has been successfully used for direct immersion SPME (DI-SPME) in grapes and strawberries [13,14,15], tomato, spinach, carrot [16], and avocado [17]. The coating could be reusable over 100 times in these complex matrices. The outstanding antifouling property of PDMS/DVB/PDMS coating facilitates quantitative analysis of more complex matrices, such as spaghetti sauce [18], pureed prune baby food [19], and edible seaweeds [20]. The analytes extend to different chemical classes, including pesticides, polycyclic aromatic hydrocarbons, polychlorinated biphenyls [20], and short-chain aliphatic amines [21]. As the matrix effect is drastically minimized or avoided, SPME can be directly combined with mass spectrometry, which dispenses with matrix pretreatment and chromatographic separation, and improves the throughput of the analytical procedure [22]. Recently, this fiber has been commercialized owing to its robust endurance for DI-SPME, which makes an important step towards in vivo/in situ analysis. Besides, there are other matrix compatible coatings [23], but little research and few applications have been reported.

SPME is a non-exhaustive extraction technique in which only a small part of analytes is removed from the sample matrix. Therefore, successful use of in vivo/in situ SPME sampling for accurate quantification of analytes requires the selection of an appropriate calibration method. Various calibration methods have been developed for in vivo analysis [24]. Among those methods, the sampling-rate ($R_{s}$) calibration [25] is particularly convenient for rapid sampling. In fact, studies on in vivo SPME became popular only after the emergence of this calibration method. This calibration assumes that the $R_{s}$ remains constant throughout the duration of sampling in the linear regimen of the extraction time profile. The relationship between the concentration of analytes in the sample matrix ($C_{s}$) and the extracted amount of analytes at time t (*n*) can be expressed with:

(1) $$C_{s}=\frac{n}{R_{s}t}$$

where $R_{s}$ is the sampling rate for the analyte and t is the sampling time. $R_{s}$ is similar between individuals within a species and independent of sampling conditions (such as temperature and concentration) [7,25]. Therefore, the sampling rates for target analytes can be determined under laboratory conditions and be directly used for quantification of analytes in real samples. The sampling rate calibration method is gaining more and more applications [6,7,9,10], but in the published literature, the calculation of concentrations is based on Equation (1) with no discussion of its feasibility and applicability when the object of study is changed. The research and development of calibration methods is far from sufficient to satisfy in vivo/in situ SPME sampling of various living organisms.

Apple, one of the most popular fruits, not only has many nutrients, but also has high medicinal value and health functions [26,27]. However, in the consumption of apples we are concerned about safety due to harmful chemicals during agricultural practices and post-harvesting. Because apples are living systems, both the beneficial compounds and harmful chemicals undergo various changes both in the natural environment and during storage and transportation. Thus, the development of in vivo/in situ monitoring method is especially useful to understand these changes and ensure food security and nutrition; so far, studies on fruits have not been reported. In this paper, a matrix-compatible PDMS/poly(styrene-co-divinylbenzene)/PDMS (PDMS/P(St-co-DVB)/PDMS) coating was produced by taking advantage of the high adsorption capacity of P(St-co-DVB) microspheres [28,29] and the antifouling properties of PDMS. Stainless steel wire was introduced in order to improve durability of the fiber instead of fused-silica when performing in situ sampling. Similar to the PDMS/DVB/PDMS fiber, the implementation of pre- and post-desorption rinsing procedures was helpful to further extend the coating lifetime. Due to the difference of apple matrix with other samples, the composition of the rinsing solutions needs to be carefully tuned. Diazinon and chlorpyrifos were chosen as model analytes as they are among the most frequently detected organophosphorus pesticides (OPPs) in apples [30,31,32]. According to the literature [24], the sampling rate calibration method should not be suitable in this study because sampling rate was changing with time, but the quantification was still realized by this method. It is a great progress to widen the application range of this calibration. Although in situ sampling was applied in this study, this method is unquestionable to be used for in vivo analysis of apples and other fruits and vegetables, both for nutrition and security monitoring.

## 2. Results and Discussion

### 2.1. Strategies to Ensure the Lifetime of the Fiber in Apple Matrix

Up to now, DI-SPME for pesticide residual investigation in apples has been rarely reported. Cherta et al. performed DI-SPME in commercial apple juice after dilution with a commercial polyacrylate (PA) fiber [33]. No significant differences or signal enhancements were observed between spiked water and spiked juice samples after 4-fold dilution with deionized water, indicating that commercial apple juice is much less complex than fresh natural apple pulp. Even so, the fiber still showed poor reproducibility at low spiking levels. The contamination of the coating cannot be neglected, especially in natural apple pulp.

In an attempt to overcome the problem with fiber fouling during extraction, a cleaning procedure including a rapid rinsing of the fiber after extraction and a post-desorption washing after desorption was implemented. Supplementary Table S1 shows the rinsing and washing conditions tested in this study. The results are given in Supplementary Figure S1. Finally, Method 5 was applied to test the endurance and reusability.

Manual cleaning of any possible debris attached to the coating surface by using a washing solvent-soaked KimWipe tissue was necessary to extend the lifetime. At first, the fiber was cleaned after each batch of 10 samples. During 100 consecutive extractions, the extraction efficiency did not show a substantial decrease, but great fluctuation was observed (Supplementary Figure S2). In most cases, the extraction ability was high in the middle of each batch. A possible explanation for this phenomenon could be that the matrices that adhered to the coating changed the coating chemistry and helped the extraction; however, too much fouling changed the surface and decreased the adsorption ability.

Further work was done with the cleaning after each sample and the usability profiles are given in Supplementary Figure S3. The relative standard deviations (RSDs) of a new fiber were 10.82% for diazinon and 9.24% for chlorpyrifos during the 85 consecutive extractions, indicating very good repeatability and a long lifetime. The RSDs were even better than those obtained by the PDMS/DVB/PDMS fiber [13]. The fiber was constantly inspected under an optical microscope. Supplementary Figure S4 shows the microscopic images of the fiber during the test. The PDMS layer bestowed a smooth surface on this new fiber. The coating turned to yellow gradually but did not drop or break. Some small black particles adhered to the surface of the coating during the extraction, but they were removed by a KimWipe tissue. The gentle wiping may have hurt the coating since the surface was rougher and rougher during the test, but it did not show much effect on the extraction ability, and the coating was still clean after 85 extractions. As a comparison, the lifetime of an uncoated PDMS/P(St-co-DVB) fiber was investigated. The RSDs of 15 consecutive extractions were 27.33% for diazinon and 32.42% for chlorpyrifos, presenting high variability and poor repeatability.

### 2.2. Stability of OPPs in Apple Matrix and Analytical Performance of the Fiber

**Figure 1:**
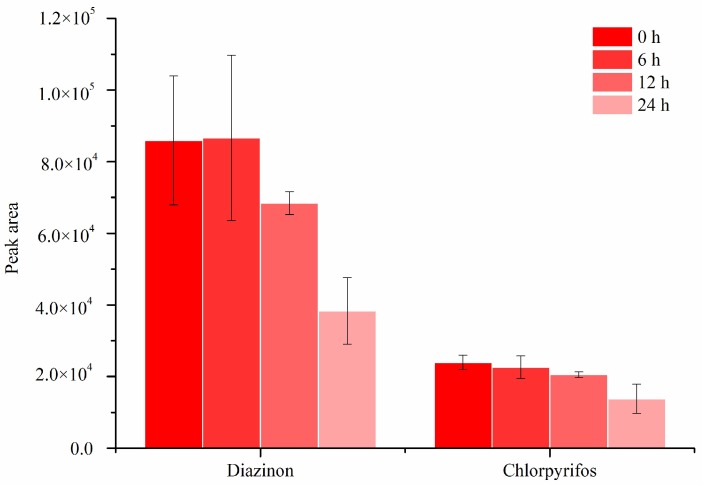
The stability of pesticides in spiked apple pulp matrix at room temperature (25 °C) with different storage times analyzed by direct immersion solid-phase microextraction/gas chromatography (DI-SPME/GC). Concentration: 125 ng/g for diazinon and 500 ng/g for chlorpyrifos. SPME conditions: extraction time, 30 min; extraction temperature, 25 °C; agitation, 600 rpm; desorption temperature, 280 °C; desorption time, 10 min.

**Figure 2:**
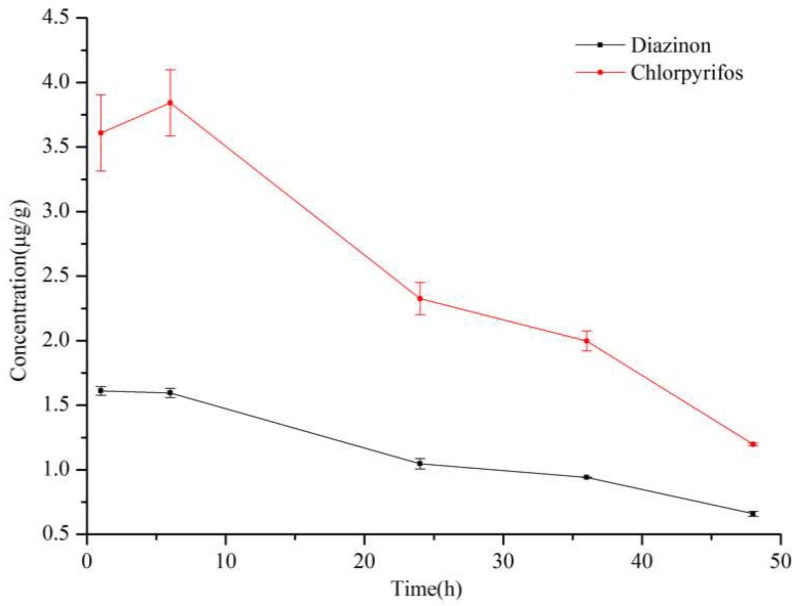
The concentrations of organophosphorus pesticides (OPPs) and their changes in peeled positive apples by the liquid–liquid extraction (LLE)/GC method. Peeled apples were soaked in 40 µg/mL of OPPs for 30 min, stored at room temperature (25 °C), and then put into analysis at different storage time.

Previous work was done for the analysis of OPPs in apple samples [34,35,36], but the stability of OPPs in the matrix has not been reported. In this study, spiked apple pulp samples after incubation for one hour were inspected by DI-SPME/gas chromatography (GC) to check the stability of OPPs in the matrix. As shown in Figure 1, the two OPPs were not stable in apple pulp and degradation occurred after 6 h. This degradation was also confirmed by the liquid–liquid extraction (LLE)/GC method for the analysis of positive apples rather than spiked pulp (Figure 2). Considering the stability from 0 to 6 h, the incubated sample was directly put into analysis without further storage.

**Figure 3:**
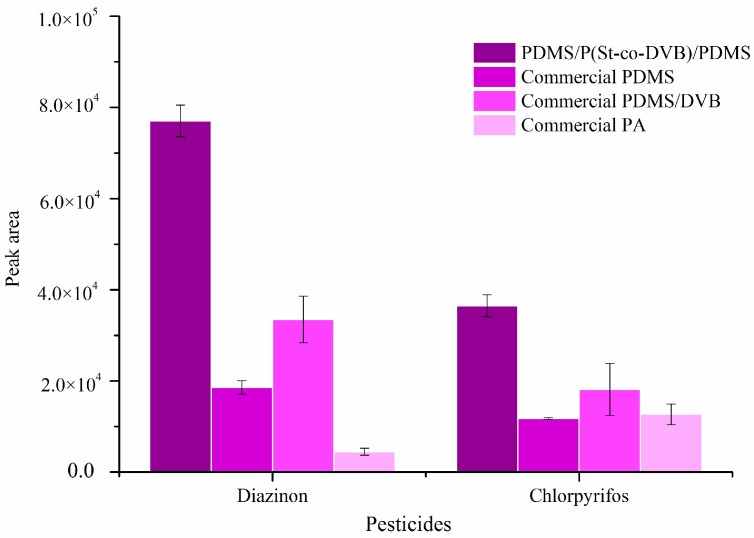
Comparison of the extraction capability of four fibers. Pesticide concentration: 6.25 ng/g for diazinon and 3.75 ng/g for chlorpyrifos. SPME conditions were the same as those in Figure 1 but the matrix was deionized water in order to protect the commercial fibers. PDMS: polydimethylsiloxane; DVB: divinybenzene; P(St-co-DVB): poly(styrene-co-divinylbenzene); PA: polyacrylate.

The extraction capacity of the prepared PDMS/P(St-co-DVB)/PDMS fiber was investigated in comparison with commercially available PA, PDMS, and PDMS/DVB fibers. The SPME parameters here were selected mainly to meet the needs of practical application of in vivo SPME for apples where salt cannot be added, temperature cannot be changed, and a long extraction time is not preferred. Nevertheless, the parameters were still close to those reported in some references for these commercial fibers [33,37]. The results shown in Figure 3 indicated that the overcoated fiber was infinitely superior to the other fibers. The high extraction ability can be ascribed to the addition of P(St-co-DVB) microspheres.

A series of standard solutions of OPPs at different concentration levels were prepared to establish calibration curves in apple pulp. The analytical performance including linearity, determination coefficient (R^2^), limit of detection (LOD), limit of quantification (LOQ), and RSD is shown in Table 1. The OPPs exhibited good linearity with satisfactory regression coefficients. The LODs were lower than the maximum residue limits of these OPPs established by the National Food Safety Standard of China (GB 2763-2016) and the European Commission (Reg.(EU) No 834/2013, 2018/686) in fruits and vegetables. The recoveries were good as the matrix was complex.

Supplementary Figure S5 shows the chromatograms of spiked and unspiked apple pulp by the DI-SPME/GC method. The coating can be used for very low levels of OPP detection.

### 2.3. Preparation of Positive Samples for In Situ Analysis

Positive apples are required in order to develop and evaluate the in situ SPME method. No apple tree is available in the city for climatic reasons. Immersion of apples in OPPs aqueous solution was taken into consideration since spraying would take a long time to absorb the OPPs. Apples were immersed in OPP solution, then taken out and hung at room temperature. Monitoring of the targets in apples was accomplished by traditional LLE/GC method at time points of 3, 4, 6, 8, and 10 d. Results showed that no OPPs were detected in peeled apples during the time range. The concentration of diazinon in whole apples was 0.42 ± 0.04 µg/g (*n* = 3) after 10 d storage, but no chlorpyrifos was found, probably because of its low enrichment and easy degradation [30]. The experiments indicated that OPPs did not go through the peel due to its wax layer. This result was consistent with some reports that OPPs were mainly concentrated in the peel of apples [32]. Punching the apples using a needle to obtain holes was also tried in order to facilitate the transportation of OPPs, but the result showed that the OPPs were not evenly distributed in the apples.

**Figure 4:**
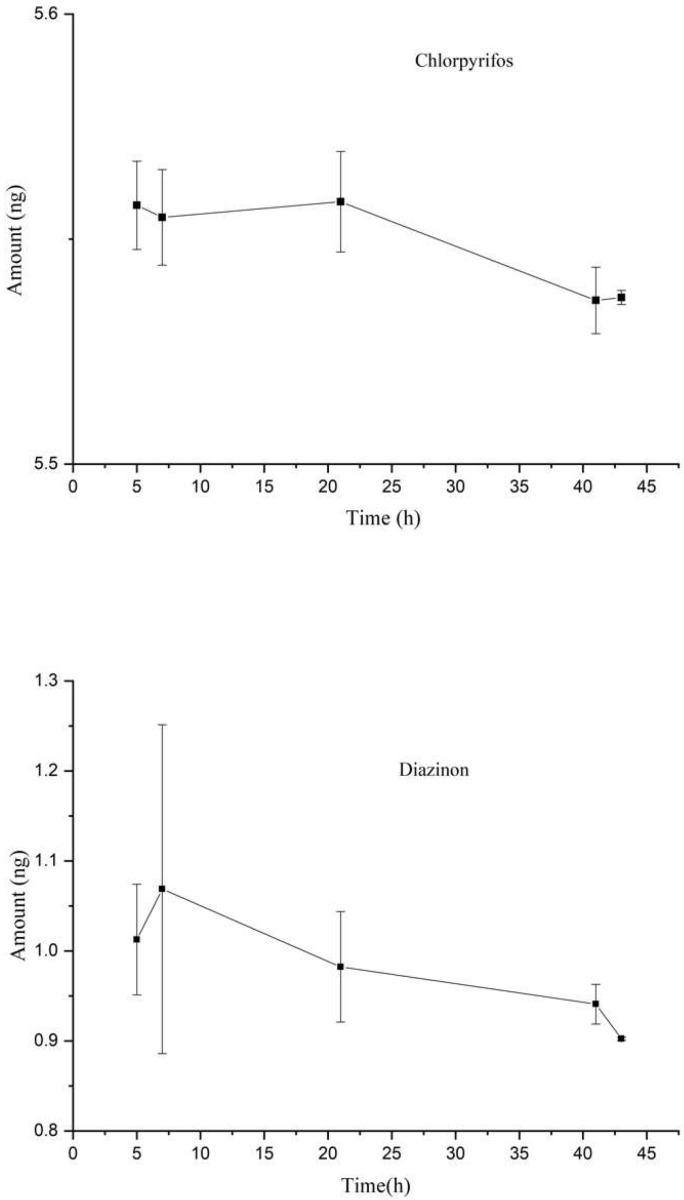
The amounts extracted by in situ SPME/GC (mean ± SD, *n* = 3) of pesticides in an apple during storage.

Peeled apples were then immersed in OPP solution. Figure 4 shows the amounts extracted by in situ SPME/GC from 5 to 43 h of storage. The amount of chlorpyrifos reduced during storage due to its migration and degradation. The amount of diazinon reached a maximum at 7 h and then decreased slowly. The repeatability was better and better as time passed. Considering the extracted amounts and repeatability, the apples after 41 h of storage were taken as positive samples for the following study. This was an alternative method since no apple trees were available. The peak areas of 45 sampling sites were obtained by in situ SPME among positive samples, and no significant statistical differences (*p* > 0.05) were found using Statistical Product and Service Solutions statistics (SPSS version 22.0) and Duncan’s multiple-range test. This indicated the OPPs existed evenly in the apple.

During the preparation of positive samples, many apples were involved and large quantities of pesticides were required, resulting in environmental pollution and high costs. At first, more pesticides including parathion-methyl and fenitrothion were investigated, but finally only chlorpyrifos and diazinon were used as representatives.

### 2.4. $R_{s}$ and Its Applicability

**Figure 5:**
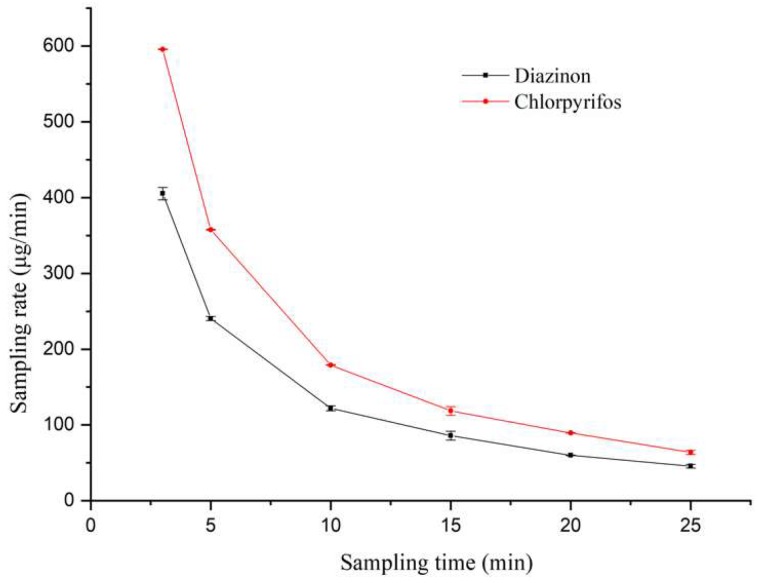
Sampling rates (mean ± SD, *n* = 5) of pesticides with different sampling durations in an apple.

According to the kinetics of the SPME process [24], immediately after the contact of the fiber with the sample, there is a rapid increase in the mass absorbed by the fiber. The rate of increase then slows and eventually reaches equilibrium. Figure 5 investigates the changes of sampling rates with sampling time. The $R_{s}$ at the 3-min sampling point was as high as 595.62 and 405.27 µg/min for chlorpyrifos and diazinon, respectively, while it declined to 63.86 and 45.60 µg/min at 25 min. The $R_{s}$ and its decline are determined by the compound itself.

It is said that the sampling rate method cannot be used in situations where sampling rate is changing with time [24], but in our study, the quantification was still achieved by this calibration method. Table 2 displays the concentrations in apples obtained by in situ SPME/GC using the $R_{s}$ calculated at their corresponding sampling time points. The detected concentrations were equal at all sampling points, indicating the applicability of the sampling rate calibration. The selection of sampling time depends on the sensitivity of the method for the investigated compound. A short sampling time is satisfying for in vivo analyses, nevertheless, a sampling time less than 30 min is acceptable in most situations.

In this study, the in situ SPME/GC method was developed to meet the need to track OPPs for a long time. Sufficient sensitivity was preferable since OPPs would experience degradation. For this reason, a sampling time of 20 min was chosen in consideration of sensitivity and sampling efficiency. At this time point, the $R_{s}$ was measured to be 69.55 ± 1.58 µg/min for diazinon and 89.81 ± 0.25 µg/min for chlorpyrifos (21 °C, room temperature). Considering the applicability of this method for in vivo sampling in natural environment, the positive apples were stored at 4 °C in a freezer, and the $R_{s}$ was 70.50 ± 2.36 µg/min for diazinon and 87.18 ± 0.14 µg/min for chlorpyrifos. The results suggested that the environmental temperatures had a negligible effect on the sampling rates, at least in a not-too-wide range. In this experiment, temperatures higher than room temperature were not tested in the case of rot of apples after storage of 41 h. In view of the fact that the optimal temperature for growing apples is between 5 °C and 25 °C, the tested range is workable.

Since the sampling rates are changing over time, it is necessary to strictly control the sampling time. Table 3 shows the effect of sampling time on the concentrations detected by in situ SPME/GC. All of the values were obtained using the same $R_{s}$ (69.55 µg/min for diazinon and 89.81 µg/min for chlorpyrifos). In the case of in situ SPME/GC, the concentrations calculated were statistically equal in the time range of 20–40 min for diazinon, and 20–25 min for chlorpyrifos. Because the $R_{s}$ was changing with sampling time, these results should be less accurate. As can be seen, the values matched well with those obtained by LLE/GC only in the range of 18 to 22 min for both of the OPPs. Theoretically, the concentrations determined by LLE/GC should be the same because the apples were treated in the same way. However, differences existed among those apples (such as water content, shape, and size), which led to the differences among the concentrations.

### 2.5. Evaluation of In Situ SPME/GC Method

Table 4 lists the LOD and LOQ obtained with the in situ SPME/GC method in positive apples with very low concentrations of diazinon and chlorpyrifos. The LOD and LOQ were estimated on the basis of a 3:1 and 10:1 signal-to-noise ratio, respectively. The LODs were rather lower than the maximum residue limits of OPPs given by the National Food Safety Standard of China (GB 2763-2016) and the European Commission (Reg.(EU) No 834/2013, 2018/686), and were similar to the LODs (0.07-2.07 ng/g) detected in aloe and cabbage [7]. The values were higher than those in Table 1 owing to different sampling conditions. The small RSDs of five replicates demonstrated the excellent repeatability of in situ SPME.

The accuracy of in situ SPME/GC quantification was verified by comparing with the concentrations determined by traditional LLE method. As presented in Table 5, the concentrations by the two methods were statistically equal (ANOVA), which demonstrated the accuracy of the proposed in situ SPME method.

### 2.6. In Situ Tracing OPPs in Apples

**Figure 6:**
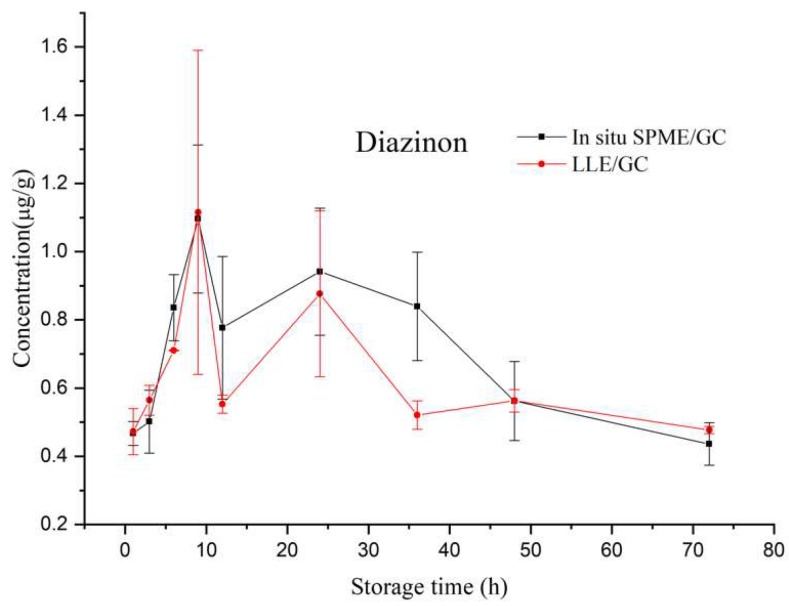

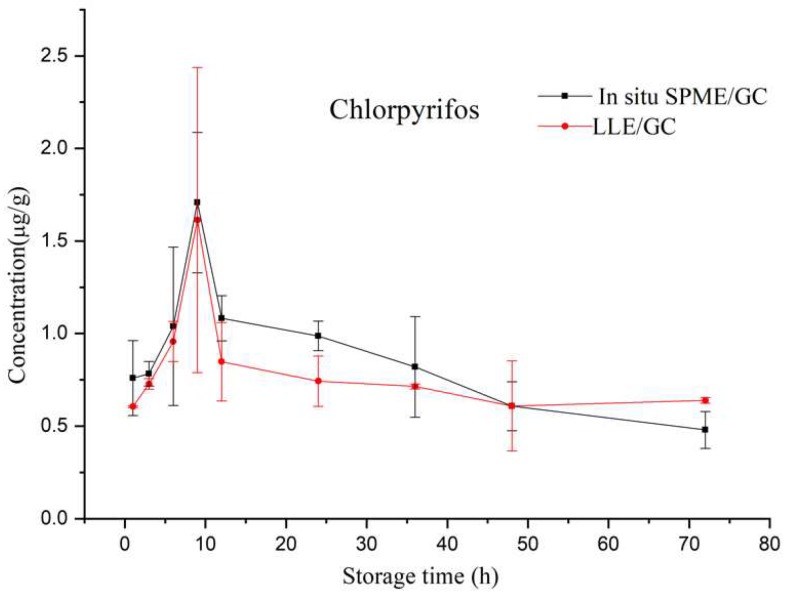
Tracing of OPPs in apples by in situ SPME/GC (*n* = 5) and LLE/GC (*n* = 3). The error bars represent the standard deviations.

In situ tracing OPPs was performed in peeled positive apples. The experiment was conducted at the time points of 1, 3, 6, 9, 12, 24, 36, 48, and 72 h. As shown in Figure 6, at the beginning of the tracing curves, a rapid rise was observed for both of the OPPs until 9 h; after the concentrations reached peak value, a fast decrease was observed, which might be caused by the biodegradation and transpiration losses [6,7], or transportation to the interior of apples; later, the rate of descent slowed down. The data achieved by the two methods were very close except at a few time points, demonstrating the excellent accuracy of in situ SPME. In general, the concentrations measured by the in situ SPME method were a little higher than those by LLE/GC before 48 h, while after 48 h, the trend was the opposite. During in situ sampling the fiber only went to a depth of 2 cm in the apple, and consequently the sample for LLE also took the similar part of apples. However, the sampling was not exactly precise, which might lead to the error. Besides, there were differences among the apples, especially for LLE, as each different apple was used at each sampling point.

**Figure 7:**
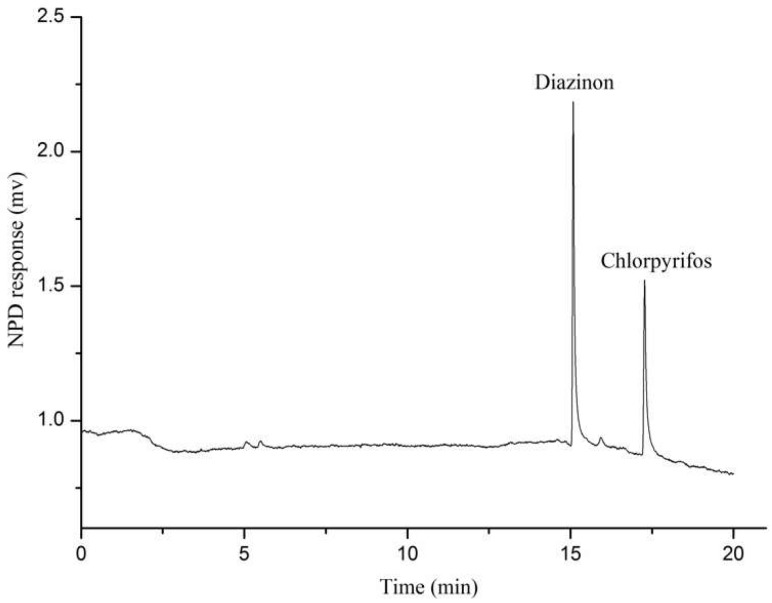
An in situ SPME/GC chromatogram of a positive apple after storage of 72 h. The concentrations of OPPs were found to be 0.436 µg/g for diazinon and 0.442 µg/g for chlorpyrifos.

Figure 7 shows an in situ SPME/GC chromatogram of a positive apple after storage of 72 h. Compared to Supplementary Figure S5, this chromatogram is “cleaner” because the matrices in an intact apple are less easily adsorbed on the coating surface. Moreover, the nitrogen and phosphorous detector (NPD) is a selective detector for nitrogen and phosphorus, which partially eliminates the interference from sample matrix.

## 3. Materials and Methods

### 3.1. Chemical Reagents, Materials, and Samples

Methanol and acetone (analytical reagent grade) were purchased from Sinopharm Chemical Reagent Co., Ltd. (Shanghai, China). Deionized water was used throughout the work. Sylgard 184^®^ (PDMS prepolymer and curing agent) was purchased from Dow Corning (Midland, MI, USA). P(St-co-DVB) microspheres (average diameter ranging from 8.0 to 9.0 μm) were purchased from Sigma-Aldrich Co., Ltd. (St. Louis, MO, USA). Stainless steel wire (80 μm o.d.) was purchased from Jinsanshun Stainless Steel Wire Co., Ltd. (Dongguan, China).

Diazinon (99%) and chlorpyrifos (99%) were purchased from J&K Chemical Technology (http://jkchemical.cn.china.cn). OPP solution with a concentration of 40 mg/mL in methanol was prepared for soaking apples in order to get positive samples.

Fuji apples from Qixia were purchased at a local market (Wuhan, China). They were first washed with deionized water, and then rinsed with nanopure water. Following, the peeled apples were soaked in 500 mL of deionized water spiked with 500 µL of OPPs solution for 30 min, then taken out and placed at room temperature for 41 h. All of the apples tested in this study were kept in good status and were similar in size and shape.

### 3.2. Instruments and Chromatographic Conditions

The chromatographic analysis was performed on a SP-6890 capillary GC system (Shandong Lunan Ruihong Chemical Engineering Instrument Co., Ltd., Tengzhou, China) equipped with a capillary split/splitless injector system and an NPD detector. Online data collection and processing were done on a chromatopac model N2000 (Hangzhou Mingtong Technology Co., Ltd., Hangzhou, China). Separations were accomplished on a SE-54 capillary column (30 m × 0.32 mm × 0.33 µm, Lanzhou ATECH technologies Co., Ltd., Lanzhou, China). Nitrogen (99.999%) was used as carrier gas at a flow rate of 12–15 cm/s for all the analyses. The temperatures of the injector and the detector were set at 280 and 290 °C, respectively. The oven temperature was initially held at 50 °C, ramped at 8 °C/min to 200 °C, and finally increased to 250 °C at a rate of 15 °C/min, held for 3 min.

A XHF-D high speed disperser (Ningbo Scientz Biotechnology Co., Ltd., Ningbo, China) was used for homogenizing the samples. A QL-861 vortex shaker (Haimen Kylin-Bell Lab Instrument Co., Ltd., Jiangsu, China) was applied for mixing the spiked samples. A magnetic stirrer DF-101S (Zhengzhou Great Wall Scientfic Industrial and Trading Co., Ltd., Zhengzhou, China) was employed for stirring the sample during extraction. An electric-heated thermostatic water bath (Changzhou Guohua Electric Appliance Co., Ltd.) was used for accelerating the solvent evaporation process during liquid extraction. The commercially available PA (85 μm), PDMS (100 μm), and PDMS/DVB (65 μm)-coated fibers were purchased from Supelco (Bellefonte, PA, USA). An Olympus TG4 digital camera (Olympus, Japan) was used for acquisition of microscopic images.

### 3.3. Preparation of SPME Fibers, SPME Procedure, LLE Procedure

See Supplementary Materials.

### 3.4. In Situ SPME

**Figure 8:**
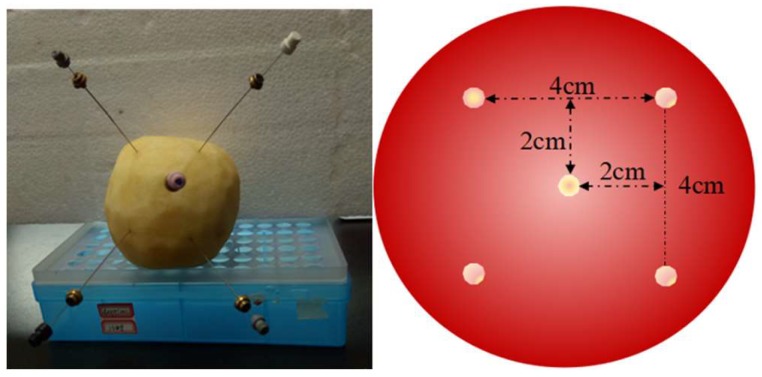
In situ SPME sampling and the schematic diagram of five sampling sites.

Apples were pierced with a 22-gauge hypodermic needle to a depth of 2 cm. A conditioned PDMS/P(St-co-DVB)/PDMS fiber was then inserted into the punched hole and reached the end. Subsequently, the fiber-protective needle was carefully withdrawn back to let the fiber exposed in the apple. After sampling, the needle was put forward to protect the fiber to a depth of 2 cm. After extraction, the fiber was rinsed for 10 s in deionized water and gently dried with a Kimwipe tissue. Then, the fiber was end sealed with silicone septum and placed at −18 °C in a freezer until desorption. After the placement of one fiber, another equal fiber was inserted the same way until five fibers were all inserted. After sampling for the same time, the five fibers were taken out following the same sequence. Figure 8 shows the in situ SPME sampling and the schematic diagram of five sampling sites. The average of the five is reported as five replicates.

## 4. Conclusions

To the best of our knowledge, this is the first tracing study of OPPs by SPME in fruits. The fiber showed excellent robustness and matrix compatibility. It is sensitive enough to be used for both in vitro and in vivo SPME analyses. The $R_{s}$ calibration assumes that the $R_{s}$ remains constant throughout the duration of sampling in the linear regimen of the extraction time profile; however, our results showed that it is still workable even if $R_{s}$ is changing with time on the condition that the sampling duration is strictly controlled. The results were accurate, the LODs were low, and the method was convenient, fast, and economical. The methodology is also promising for the studies of both beneficial compounds and harmful chemicals in fruits and vegetables to ensure their nutrition and safety. To reach this goal, matrix-compatible coatings with powerful extraction coverage of analytes are expected, and the feasibility and applicability of calibration methods need to be investigated so as to extend their application range. Of course, new calibration methods are especially welcomed.

## Figures and Tables

**Table 1:** Evaluation of the DI-SPME/GC method in apple pulp.

Analyte	Linear Range (ng/g)	R^2^	LOD ^a^ (ng/g)	LOQ ^b^ (ng/g)	RSD ^c^ (%)	Spiking Level (ng/g)	Recovery (%)
Diazinon	1.25–25	0.9996	0.02	0.07	8.11	2.5	89.90 ± 4.89
						12.5	86.31 ± 9.21
						25	87.56 ± 11.96
Chlorpyrifos	1.25–250	0.9991	0.10	0.33	4.08	12.5	74.82 ± 6.64
						25	90.02 ± 8.91
						125	98.50 ± 8.84

^a^ The LOD and ^b^ LOQ were obtained based on the standard deviation (δ) of the responses in six replicated blank extractions and the slope (S) of the calibration curve according to the equations LOD = 3 δ/S and LOQ = 10 δ/S. ^c^ Spiked concentrations: 12.5 ng/g for diazinon and 25 ng/g for chlorpyrifos. LOD: limit of detection; LOQ: limit of quantification; RSD: relative standard deviation.

**Table 2:** Comparison of concentrations (mean ± SD, *n* = 5) of pesticides obtained by in situ SPME in apples at different sampling times and their sampling rates.

Analyte	Sampling Time (min)	Concentration (µg/g)	RSD%
Diazinon	3 10 20	1.61 ± 0.04 1.70 ± 0.11 1.64 ± 0.06	4.92
Chlorpyrifos	3 10 20	2.10 ± 0.01 2.10 ± 0.01 2.10 ± 0.01	0.30

**Table 3:** Concentrations (mean ± SD) in apples obtained by in situ SPME/GC (*n* = 5) and LLE/GC (*n* = 3).

Analyte	t (min)	Concentration (µg/g)		RSD%
		In situ SPME/GC	LLE/GC	
Diazinon	15	1.62 ± 0.28 ^a^	2.62 ± 0.31	29.21
	18	2.02 ± 0.48 ^b^	2.07 ± 0.12	1.7
	20	1.26 ± 0.16 ^c^	1.24 ± 0.10	0.95
	22	1.19 ± 0.16 ^c^	1.36 ± 0.03	9.25
	25	1.12 ± 0.16 ^c^	1.54 ± 0.01	22.24
	30	1.02 ± 0.39 ^c^	1.73 ± 0.12	38.54
	40	0.90 ± 0.30 ^c^	1.64 ± 0.03	39.65
Chlorpyrifos	15	4.20 ± 0.31 ^a^	5.09 ± 0.56	12.38
	18	5.02 ± 0.25 ^b^	5.06 ± 0.39	0.59
	20	3.62 ± 0.12 ^c^	3.25 ± 0.02	8.87
	22	3.42 ± 0.09 ^c^	3.58 ± 0.09	3.39
	25	3.12 ± 0.11 ^c^	3.82 ± 0.10	14.4
	30	2.51 ± 0.14 ^d^	3.92 ± 0.22	30.26
	40	1.95 ± 0.19 ^e^	4.05 ± 0.11	40.75

The letters a, b, c, d, and e indicate that there are differences between the values by ANOVA (*p* < 0.05).

**Table 4:** LOD, LOQ, and RSD of in situ SPME/GC for OPPs in apples.

Analyte	LOD (ng/g)	LOQ (ng/g)	RSD%
Diazinon	0.18	0.60	2.28
Chlorpyrifos	0.20	0.67	0.28

**Table 5:** Comparison of concentrations (mean ± SD) obtained by in situ SPME/GC (*n* = 5) and LLE/GC (*n* = 3) in apples with different soaking concentrations.

Analyte	Soaking Concentration (µg/mL)	Concentration (µg/g)		RSD%
		In Situ SPME/GC	LLE/GC	
Diazinon	20	1.32 ± 0.02	1.13 ± 0.01	7.9
	40	1.58 ± 0.12	1.75 ± 0.01	7.75
	60	1.64 ± 0.02	1.84 ± 0.09	6.04
Chlorpyrifos	20	2.73 ± 0.01	2.22 ± 0.02	9.79
	40	2.80 ± 0.02	2.88 ± 0.32	6.22
	60	2.62 ± 0.02	2.85 ± 0.09	4.91

## Supplementary Materials

The following are available online at https://www.mdpi.com/1420-3049/24/24/4444/s1. Table S1: Rinsing and washing conditions tested in this study. Figure S1: Peak areas of sequential extractions with different rinsing and washing conditions, including (a) Method 1, (b) Method 2, (c) Method 3, (d) Method 4, and (e) Method 5. Figure S2: Reusability profiles of a PDMS/P(St-co-DVB)/PDMS fiber subjected to 100 DI-SPME in apple pulp for diazinon (a) and chlorpyrifos (b). Normalized response was calculated as the ratio between the amounts extracted at each extraction and the first extraction. Figure S3: Reusability profiles of a PDMS/P(St-co-DVB)/PDMS fiber in apple pulp. Normalized response was calculated as the ratio between the amounts extracted at each extraction and the first extraction. Figure S4: Microscopic images of a PDMS/P(St-co-DVB)/PDMS fiber before extraction (a), after 10 extraction cycles in apple pulp (b1) and cleaning (b2), after 50 extraction cycles in apple pulp (c1) and cleaning (c2), and after 85 extraction cycles in apple pulp (d1) and cleaning (d2). Figure S5. Chromatograms of a real sample and a spiked sample using DI-SPME/GC method.
