# Supplementary material for: In Situ Real-Time Tracing of Organophosphorus Pesticides in Apples by Solid-Phase Microextraction with Developed Sampling-Rate Calibration
Source: Molecules. 2019 Dec 4;24(24):4444. doi: 10.3390/molecules24244444 (PMC6943702; doi:10.3390/molecules24244444)
Supplement: Supplementary file 1 [file molecules-24-04444-s001.pdf]

Article

In Situ Real-Time Tracing of Organophosphorus Pesticides in Apples by Solid-Phase Microextraction with Developed Sampling-Rate Calibration

Xiao-Fan Zhang [1,†], Li-Li Zhao [1,2,†], Ming-Quan Huang [3], Xiu-Juan Li [1,*] and Si-Yi Pan [1]

[1] Key Laboratory of Environment Correlative Dietology (Ministry of Education), College of Food Science & Technology, Huazhong Agricultural University, Wuhan 430070, China; xiaofanzhang2010@163.com (X.-F.Z.); hzauzjy@163.com (L.-L.Z.); pansiyi@mail.hzau.edu.cn (S.-Y.P.);

[2] Institute of Agricultural Products Processing, Henan Academy of Agricultural Sciences, Zhengzhou 450002, China

[3] China Light Industry Key Laboratory of Liquor Quality and Safety, Beijing Technology and Business University, Beijing 100048, China; huangmq@th.btbu.edu.cn

* Correspondence: lixiujuan@mail.hzau.edu.cn; Tel.: +86-27-8728-2111

† These authors contributed equally to this paper.

Supplementary Materials

Table S1. Rinsing and washing conditions tested in this study.

Method	Conditions
1	Rinsing for 10 s in deionized water
	Washing in deionized water for 2 min
	Apple: deionized water =1:1 (*w*/*w*)
2	Rinsing for 30 s in deionized water
	Washing in water: methanol=1:1 (*w*/*w*) for 2 min
	Apple: deionized water =1:1 (*w*/*w*)
3	Rinsing for 10 s in water:methanol (*w*/*w*) =9:1
	Washing in water: methanol(*w*/*w*)=1:1 for 1min
	Apple: deionized water =1:1 (*w*/*w*)
4	Rinsing for 10s in water:methanol (*w*/*w*)=9:1
	Washing in methanol: acetone(*v*/*v*)=1:1 for 1min
	Apple: deionized water =1:1 (*w*/*w*)
5	Rinsing for 10s in water:methanol(*w*/*w*)=9:1
	Washing in methanol: acetone(*v*/*v*)=1:1 for 1min;
	Apple: deionized water =1:2 (*w*/*w*)

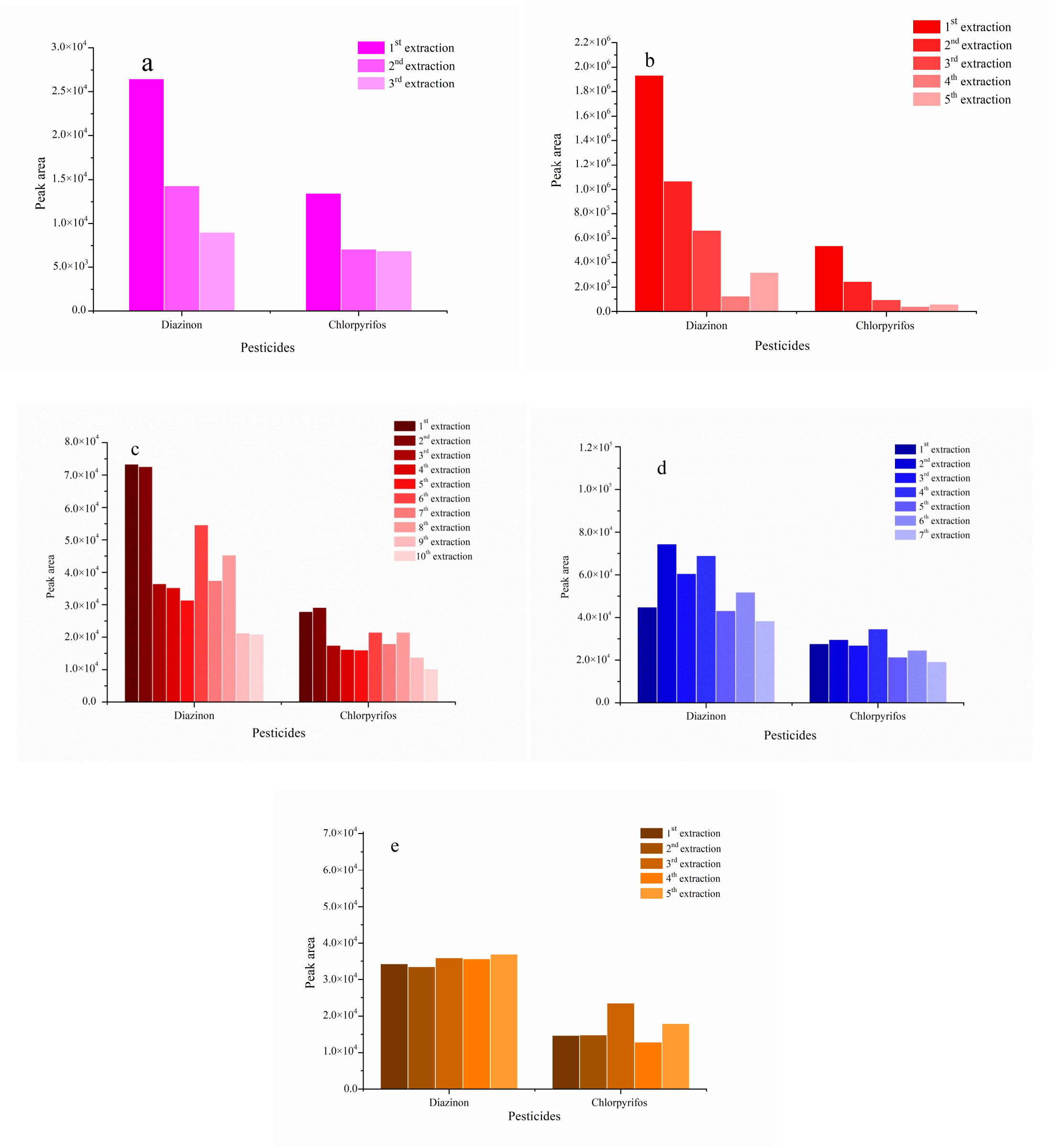

Figure S1. Peak areas of sequential extractions with different rinsing and washing conditions, including (**a**) Method 1, (**b**) Method 2, (**c**) Method 3, (**d**) Method 4, and (**e**) Method 5.Pesticide concentration: 5 ng/gfor diazinon and 62.5 ng/gforchlorpyrifos.

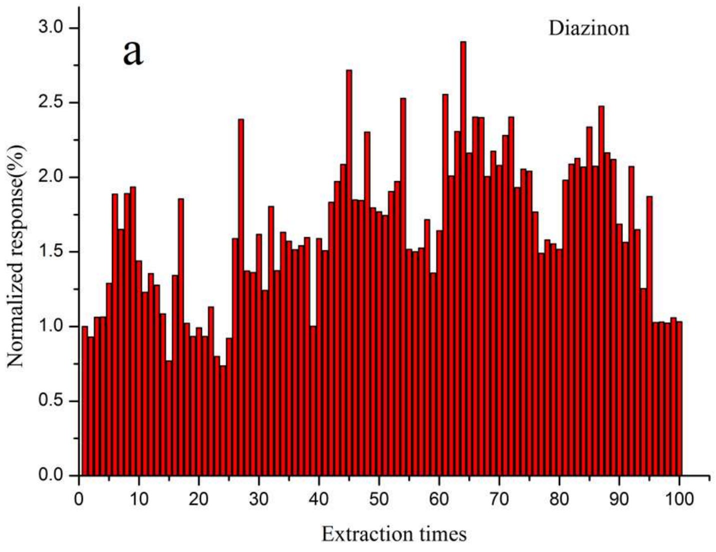

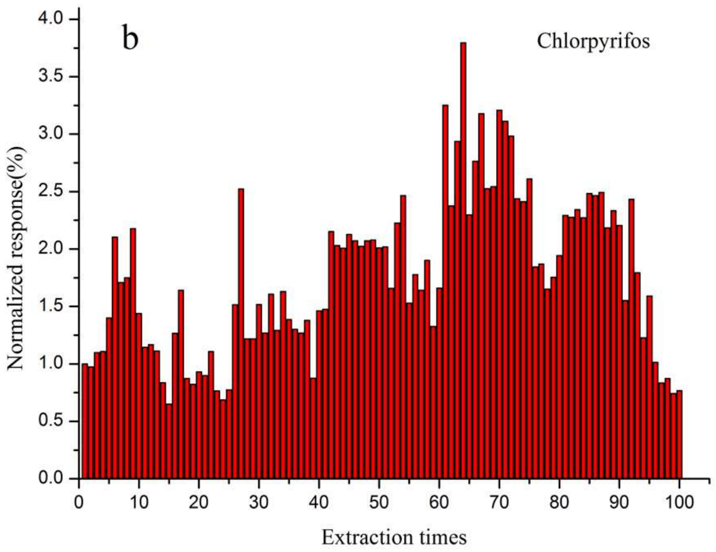

Figure S2. Reusability profiles of a PDMS/P(St-co-DVB)/PDMS fiber subjected to 100 direct immersion solid-phase microextraction(DI-SPME) in apple pulp for diazinon (**a**) and chlorpyrifos (**b**). Normalized response was calculated as the ratio between the amounts extracted at each extraction and the first extraction. Pesticide concentration: 5 ng/gfor diazinon and 62.5 ng/gfor chlorpyrifos. PDMS: polydimethylsiloxane; P(St-co-DVB): poly(styrene-co-divinylbenzene).

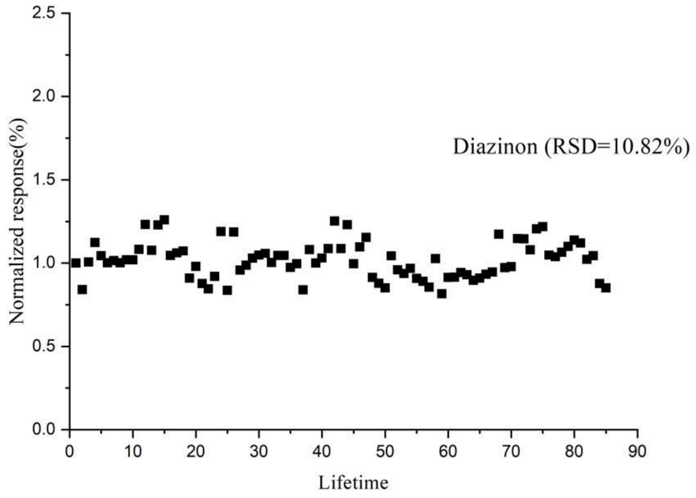

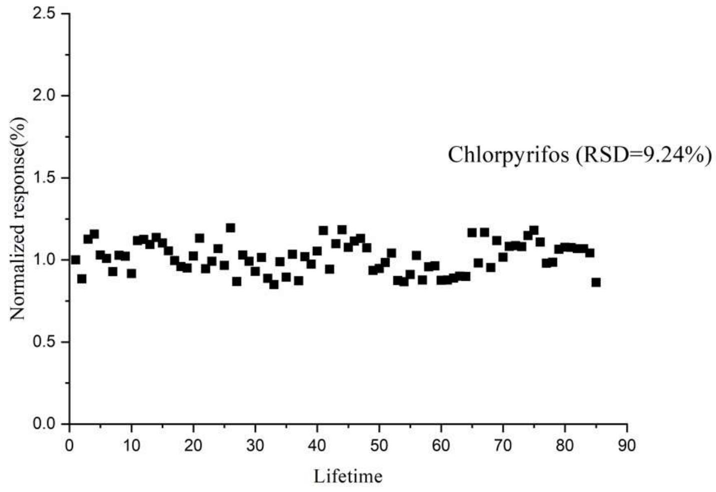

Figure S3. Reusability profiles of a PDMS/P(St-co-DVB)/PDMS fiber in apple pulp. Normalized response was calculated as the ratio between the amounts extracted at each extraction and the first extraction. Pesticide concentration: 5 ng/gfor diazinon and 62.5 ng/gfor chlorpyrifos. RSD: relative standard deviation.

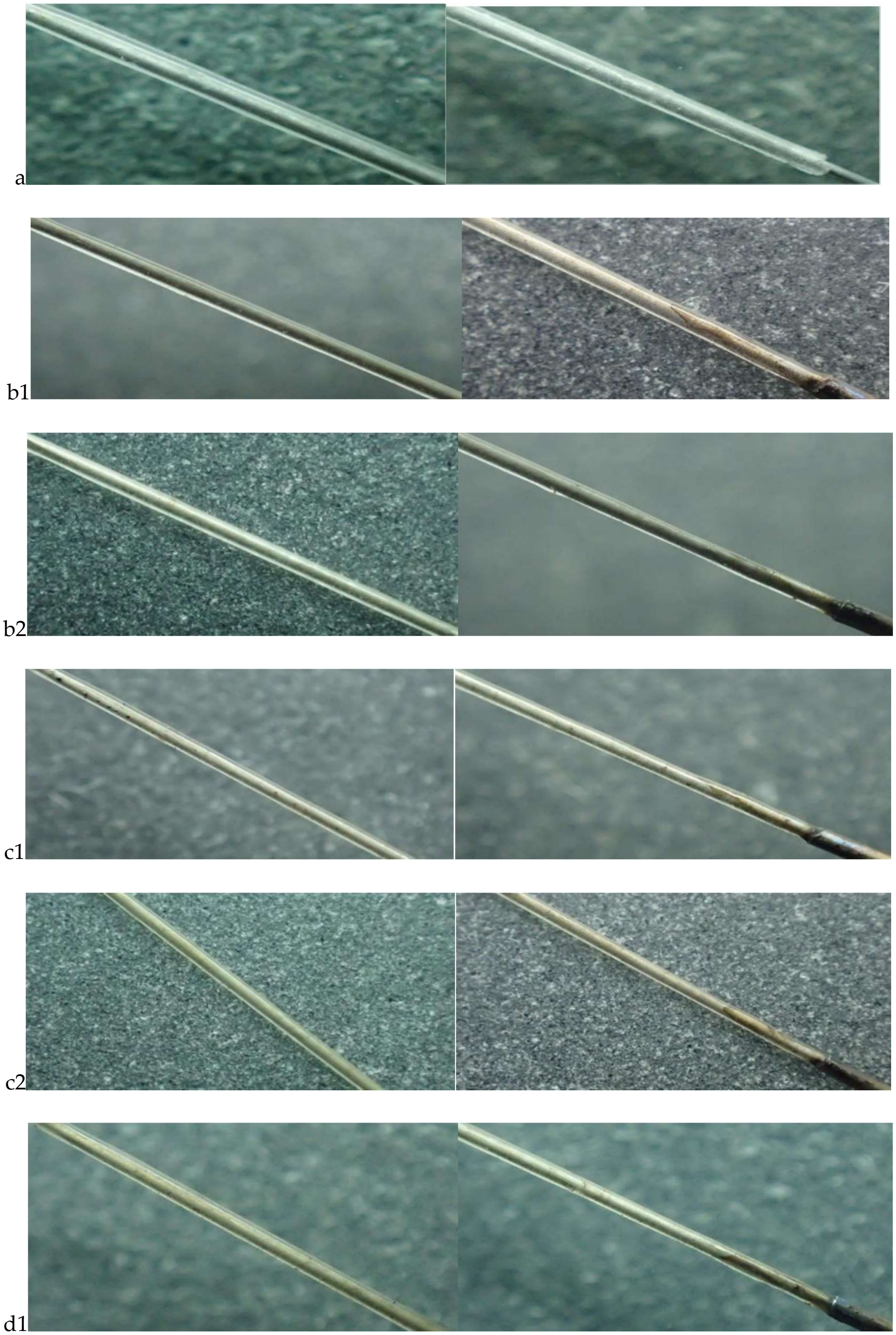
a
b1
b2
c1
c2
d1

d2
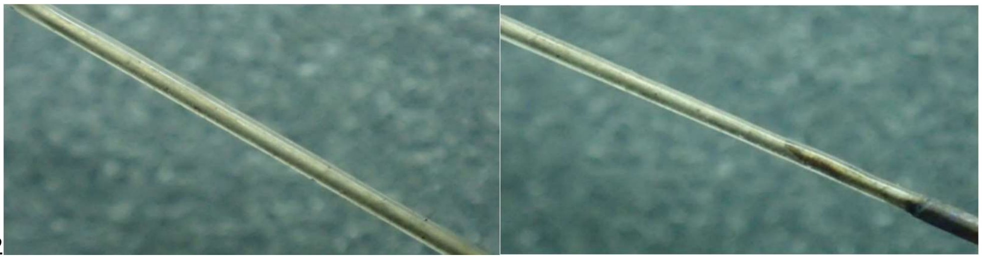

Figure S4. Microscopic images of a PDMS/P(St-co-DVB)/PDMS fiber before extraction (**a**), after 10 extraction cycles in apple pulp (**b1**) and cleaning (**b2**), after 50 extraction cycles in apple pulp (**c1**) and cleaning (**c2**), and after 85 extraction cycles in apple pulp (**d1**) and cleaning (**d2**).The left images are the coatings near the fiber tip and the right images are the coatings near the connector to the SPME handle. The magnification factor was 16.

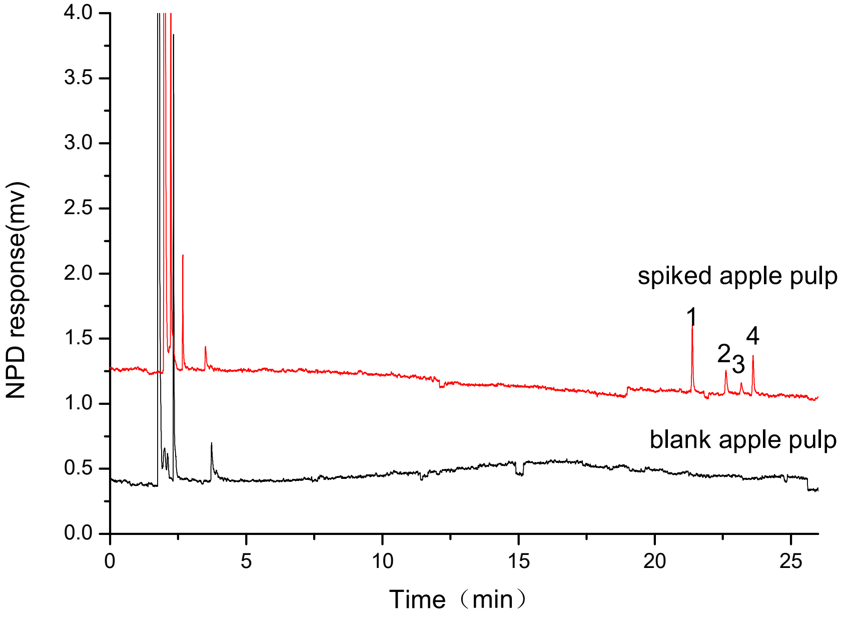

Figure S5. Chromatograms of a real sample and a spiked sample using DI-SPME/gas chromatography method. Peak: 1, diazinon; 2, parathion-methyl; 3, fenitrothion; 4, chlorpyrifos. Spiking levels: A, diazinon and fenitrothion, 2.5 ng/g; parathion-methyland chlorpyrifos,12.5 ng/g.

Preparation of SPME Fibers

Sylgard 184 PDMS prepolymer and curing agent were mixed thoroughly at a ratio of 10:1, according to the manufacturer's manual, in a polypropylene centrifuge tube, and subjected to centrifugation for 8 min at 12000 rpm for degassing. A portion of 45 mg of P(St-co-DVB) microspheres was mixed into 700 mg of the resulted PDMS solution to obtain uniform PDMS/P(St-co-DVB) mixture. A corroded stainless steel wire was dipped vertically into the mixture and subsequently pulled out at a slow rate, and a PDMS/P(St-co-DVB) coating was formed on the surface of the wire end (about 1.5 cm). The coated fiber was placed in a drying oven at 150 °C for 2 min to cure the coating. For each fiber, the coating/curing process was repeated to obtain appropriate thickness. The PDMS overcoated PDMS/P(St-co-DVB) fibers were prepared according to the previous study by Souza Silva [13]. PDMS fibers without P(St-co-DVB) microspheres were also prepared for daily quality control (QC) tests. The coatings with thickness of 59 μm and length of 1.5 cm were selected in this study.

Prior to use, the fibers were conditioned in a GC injection port under inert gas flow (N_2) at 250 °C for 2 h, and then 280 °C for 1 h. During the usage, the coatings were inspected using an optical microscope.

SPME Procedure

After chopping and removing the cores, the apple was homogenized along with 2-fold amount of deionized water using a high speed disperser. The processed sample was placed in amber glass bottles and stored in a freezer at 4 °C until analysis.

DI-SPME was carried out in 10-mL vials sealed with Teflon-coated septum sealed steel caps. After being spiked with pesticides, the apple pulp was agitated for one hour at 600 rpm and 30 °C before further pretreatment in order to allow for the sufficient binding of the analytes to the matrix. Each portion of 8 g of apple pulp was weighed in a 10-mL vial. A 15-min pre-extraction incubation of the sample was performed in the agitation unit at 600 rpm and 25 °C, followed by 30 min of extraction at the same temperature and stirring rate. Following extraction, the fiber was rinsed in a rinsing solvent, desorbed for 10 min at 280 °C, and then washed with a washing solvent. After each sample, a KimWipe tissue soaked with the washing solvent was used manually to gently clean the fiber surface of any attached debris. All experiments were performed in triplicate.

For the evaluation of DI-SPME/GC method, the extraction temperature was 30 °C, the extraction time was 50 min, the stirring rate was 1200 rpm and the desorption time was 5 min. Other conditions were the same as above.In this part, all experiments were performed in quintuplicate.

The stability of the instrumental response was monitored by QC tests, which were conducted by a home-made PDMS fiber in 8 mL of spiked deionized water at the beginning of each working day.

LLE Procedure

Organophosphorus pesticides (OPPs) in apple samples were extracted and purified according to the Agricultural Standard of China NY/T761-2008 (http://down.foodmate.net/standard/sort/5/15091.html).

Duringin situ sampling the fiber only went to a depth of 2 cm in the apple, and the sample for LLE also took the similar part of apple.

Briefly, a portion of 20 g of chopped apple sample was homogenized along with 50 mL of acetonitrile using a high speed disperser, and then the mixture was vigorously shaken for 5 min and filtrated by suction filtration. The filtrate with the volume of 40-50 mL was collected in a 100-mL polypropylene centrifugal tube. Subsequent phase separation was facilitated by adding 6 g of sodium chloride and shaking for 1 min. After centrifugation for 15 min at 4000 rpm, 10 mL of acetonitrile solution from the upper layer was suctioned out and put into a 50-mL polypropylene centrifugal tube and then evaporated to near dryness in water bath under 80 °C with a steam of nitrogen. Less than 2 mL of methanol was applied to flush the tube two times, and the solutions were totally transferred

to a 2-mL volumetric flask. Finally the solution was diluted to 2 mL with methanol and mixed thoroughly by up and down movements. A portionof1 μL wasinjectedinto GC systemforanalysis.
